# Characterising four *Sarconesiopsis magellanica* (Diptera: *Calliphoridae*) larval fat body-derived antimicrobial peptides

**DOI:** 10.1590/0074-02760200587

**Published:** 2021-07-16

**Authors:** Cindy Pérez, Andrea Díaz-Roa, Yuly Bernal, Nelson E Arenas, Dario Eluan Kalume, Luzia Monteiro de Castro Côrtes, Pedro I da, Yahson Varela, Manuel A Patarroyo, Orlando Torres, Felio J Bello

**Affiliations:** 1 Universidad Antonio Nariño, Facultad de Medicina Veterinaria, Bogotá, Colombia; 2 Instituto Butantan, Laboratório de Toxinología Aplicada, São Paulo, SP, Brasil; 3 Universidad Nacional Abierta y a Distancia, Escuela de Ciencias Agrícolas, Pecuarias y de Medio Ambiente, Bogotá, Colombia; 4 Universidad Antonio Nariño, Facultad de Ciencias, Bogotá, Colombia; 5 Fundação Oswaldo Cruz-Fiocruz, Instituto Oswaldo Cruz, Laboratório Interdisciplinar de Pesquisas Médicas, Rio de Janeiro, RJ, Brasil; 6 Fundação Oswaldo Cruz-Fiocruz, Instituto Oswaldo Cruz, Laboratório de Biologia Molecular e Doenças Endêmicas, Rio de Janeiro, RJ, Brasil; 7 Fundación Instituto de Inmunología de Colombia, Molecular Biology and Immunology Department, Bogotá, Colombia; 8 Universidad Nacional de Colombia, Faculty of Medicine, Microbiology Department, Bogotá, Colombia; 9 Universidad Santo Tomás, Health Sciences Division, Bogotá, Colombia; 10 Universidad de La Salle, Facultad de Ciencias Agropecuarias, Programa de Medicina Veterinaria, Bogotá, Colombia

**Keywords:** Sarconesiopsis magellanica, antimicrobial peptide, larval fat body, physicochemical characterization, antibacterial evaluation

## Abstract

**BACKGROUND:**

The inappropriate use of antibiotics has led to the accelerated growth of resistance to antibiotics. The search for new therapeutic strategies (i.e., antimicrobial peptides-AMPs) has thus become a pressing need.

**OBJECTIVE:**

Characterising and evaluating *Sarconesiopsis magellanica* larval fat body-derived AMPs.

**METHODS:**

Fat body extracts were analysed by reversed-phase high-performance liquid chromatography (RP-HPLC); mass spectrometry was used for characterising the primary structure of the AMPs so found. ProtParam (Expasy) was used for analysing the AMPs’ physico-chemical properties. Synthetic AMPs’ antibacterial activity was evaluated.

**FINDINGS:**

Four new AMPs were obtained and called sarconesin III, IV, V and VI. Sarconesin III had an α-helix structure and sarconesins IV, V and VI had linear formations. Oligomer prediction highlighted peptide-peptide interactions, suggesting that sarconesins III, V and VI could form self-aggregations when in contact with the microbial membrane. AMPs synthesised from their native molecules’ sequences had potent activity against Gram-positive bacteria and, to a lesser extent, against Gram-negative and drug-resistant bacteria. Sarconesin VI was the most efficient AMP. None of the four synthetic AMPs had a cytotoxic effect.

**MAIN CONCLUSIONS:**

*S. magellanica* larval fat body-derived antimicrobial peptides are an important source of AMPs and could be used in different antimicrobial therapies and overcoming bacterial resistance.

The unregulated, excessive and improper use of antibiotics and their unregulated sale has undoubtedly led to a persistent increase in antimicrobial resistance (AMR), one of the greatest problems regarding world health threatening the ability to successfully treat bacterial infections.^1^ AMR is currently associated with high morbidity and mortality in humans and animals.^2^ The World Health Organisation’s (WHO) Global Antimicrobial Resistance Surveillance System (GLASS) has analysed data from 49 countries, finding high levels of antimicrobial resistance regarding bacteria such as *Escherichia coli*, *Klebsiella pneumoniae, Salmonella* spp*., Acinetobacter* spp*.*, *Staphylococcus aureus* and *Streptococcus pneumoniae*. It has been estimated that around 10 million people could die every year from such resistance by 2050 (www.who.int). The search for new therapeutic strategies (i.e., antimicrobial peptides) has thus become necessary and urgent for treating bacteria and dealing with such microorganisms’ mechanisms of action (MoA) and resistance against conventional antibiotics.

Antimicrobial peptides (AMPs) are active against Gram-negative and/or Gram-positive bacteria, fungi, parasites and viruses;^3^ some AMPs have been reported to modulate alternative cell functions such as apoptosis, wound healing and immunological response.^4^

AMPs have been isolated and characterised from larval excretions and secretions (ES), haemolymph, salivary glands and fat body derived from necrophagous flies from the Calliphoridae family, e.g., lucifensin (*Lucilia sericata*),^5^ lucifensin II (*L. cuprina*)^6^ and FLIP7 (*Calliphora vicina*).^7^ Sarconesin and sarconesin II have recently been obtained from *Sarconesiopsis magellanica* larval ES^8^ ^,^ ^9^ and shown to have accion against a broad range of bacteria such as *Streptococcus* spp., *S. epidermidis*, *Enterococcus faecalis*, *E. coli*, *Pseudomonas aeruginosa*, *Micrococcus luteus* and *S. enterica*.

Insect-derived AMPs are mainly produced in the fat body and produced in small concentrations; however, the presence of pathogens increases their concentration and they become transported to the haemolymph and other tissues.^10^ The fat bodies have an important function regarding the innate immune system;^11^ they express specific peptidoglycan recognition receptors. The Toll and immune deficiency (IMD) pathways become activated after binding to such receptors, resulting in systemic AMP expression and secretion (i.e., drosomycin, diptericin, attacin pathways).^12^ The insects’ fat bodies are analogous to the adipocytes in vertebrates’ livers; these consist of mesodermal cells called trophocytes or adipocytes.^13^ The fat body plays a major role during an insect’s metamorphosis, regulating metabolism, storing energy in the form of lipids, glycogen and proteins.^13^ ^,^ ^14^

*Sarconesiopsis magellanica (*Diptera: Calliphoridae) is a medically and forensically important blowfly having necrophagous feeding habits. Our previous studies have shown that *S. magellanica*-derived larval ES have proved effective in treating diabetic rabbits’ wounds and as potential therapy against bacterial pathogens.^8^ ^,^ ^9^ ^,^ ^15^ Such situation becomes even more drastic regarding hard-to-heal wounds in patients having an underlying disease, including diabetes or cardiovascular failure, often involving polymicrobial colonisation by different bacterial strains, such as *P. aeruginosa*, *S. pyogenes*, *Clostridium perfringens*, *Corynebacterium* spp., *Propionibacterium* spp., *S. aureus* and methicillin-resistant *S. aureus* (MRSA), or in patients in intensive care units (ICU) who have developed a health-care associated-infection (HAI).^16^

These ideas have been addressed regarding the detection of new biological properties in *S. magellanica*-derived AMPs. The need to study their inhibitory effect on bacterial pathogens directed our aim towards characterising four new *S. magellanica* larval fat body-derived AMPs.

## MATERIALS AND METHODS

*Establishing and maintaining S. magellanica colonies* - Adult specimens were collected in Bogota’s National Park Enrique Olaya Herrera (Colombia: 4º37′16″N 74º03′35″W), based on Colombian Ministry for the Environment and Sustainable Development regulatory framework (Resolution 0922, 15th May 2017) for collecting and gaining access to genetic resources. The captured specimens were taken to the Universidad Antonio Nariño’s Entomology Laboratory (Bogotá, Colombia) and kept in 25 X 25 X 25 cm entomological cages in controlled laboratory conditions: 25ºC ± 2, 60% relative humidity and 12/12 h photoperiod. The adults were fed on liver as protein source and sugar water as carbohydrate supplement. The eggs were transferred to glass flasks containing the same protein substrate where stage I larvae hatched and continued their biological development until the pupal phase. These were then separated into other sand substrate-containing flasks, thereby guaranteeing blowfly life-cycle continuity. Sterile stage III larvae were used as biological raw material for fat body extraction.

*Extracting fat bodies* - Around 200 stage III *S. magellanica* larvae were used for every experiment, following a described previously protocol;^15^ the larvae were pre-immunised by submerging them for 1 h in a 1.5 x 10^8^ CFU/mL bacterial suspension consisting of *S. aureus* and *E. coli*. After incubation, the larvae were disinfected using a 0.05% sodium hypochlorite solution for 5 min, followed by 5% formaldehyde for 5 min and three successive washes of 3 min each using sterile distilled water.^15^ The larvae’s oral region was removed, together with the salivary glands and stomachs. Larval content was extracted and fat bodies placed into 2 mL tubes containing 1 mL 2 M cold acetic acid; this was homogenised and lyophilised in a porcelain mortar. The sample was dissolved in 1,000 μL acetonitrile-water (ACN-H20) (50:50), acidified with 0.5% trifluoroacetic acid (TFA), stirred for 30 min, vortexed for 10 min and spun at 10,000xg for 30 min at 4ºC; 1 mL (ACN: 0.5% TFA) and 5 mL (0.1% TFA) were added to the supernatant for every mL recovered.^5^ ^,^ ^6^

*Peptide preparation and purification* - Sample filtration was carried out by using an Amicon Ultra-15 Centrifugal membrane for separating 10 kDa molecular weight molecules. Fat body extract was spun at 4,200 g for 10 min at 5ºC. Filtrate containing <10 kDa molecules was used for this study; 40 μL was used for quantifying protein by the bicinchoninic acid (BCA) method, 10 μL for evaluating antibacterial activity against *S. aureus* ATCC 6538 and *E. coli* ATCC 26922 whilst the remaining fat body filtrate was lyophilised.

The < 10 kDa (132 mg) filtrate was dissolved in 850 μL solution A (water: 0.05% TFA) and 150 μL solution B (ACN: 0.05% TFA) and fractionated by reversed-phase high-performance liquid chromatography (RP-HPLC) (Merck-Hitachi semi-preparative equipment), using a Kromasil C18 HPLC column (Sigma-Aldrich: 10 μm, 10 mm x 250 μm), with a 0-60% ACN/water/TFA solvent gradient for 70 min at a 3 mL/min flow rate. The chromatographic fractions were monitored at 210 nm and manually collected, lyophilised and suspended in 100 μL sterile deionised water for protein quantification and evaluating antibacterial activity.

A C18 reversed phase HPLC column (Phenomenex: 4.6 μm, 25 mm, 5 μm) was used for analysing fractions having antibacterial activity. Each fraction was dissolved in solution A and eluted with solution B having a 0%-70% linear gradient for 40 min at 1 mL/min flow rate and monitored at 210 nm. The fractions were manually collected, lyophilised and dissolved in 100 μL sterile deionised water for evaluating antibacterial activity and analysed by mass spectrometry (MS/MS).^6^

*Protein quantification* - The bicinchoninic acid assay (BCA) method was used for quantifying the proteins using a NanoDrop 2000c full-spectrum, UV-Vis spectrophotometer (Thermo Scientific). Pierce BCA Protein Assay Kit instructions were followed for preparing the bovine serum albumin standards and samples. The tubes were incubated at 37ºC for 30 min.

*MS/MS spectrometry* - The purified fractions were analysed by liquid chromatography coupled to MS (LC-MS/MS), using a hybrid mass spectrometer (LTQ-Orbitrap Velos, Thermo Fisher Scientific Waltham, MA, USA) coupled to an EASY-nLCII nano liquid chromatography (nanoLC) system (Thermo Fisher Scientific Inc). A 5 μL volume of each sample was loaded into a Jupiter C-18 pre-column (10 μm, 100 μm I.D. x 50 mm) (Phenomenex Inc., Tirrance, CA, USA) coupled to an ACQUA C-18 analytical reversed phase column (5 μm, 75 μm I.D. x 100 mm) (Phenomenex Inc). Electrospray ionisation voltage was set at 2.0 kV and temperature source at 200ºC; all MS data was acquired in positive ion mode. Fourier transform mass spectrometry (FTMS) was used for acquiring mass spectra; full scan (MS1) involved using 200-2,000 m/z (60,000 resolution at 400 m/z) as mass scan interval with the instrument operating in data-dependent acquisition mode. The five most intense ions per scan were selected for collision-induced dissociation (CID) fragmentation. The minimum signal required for selecting an ion for a fragmentation event (MS2) was set at 5,000 cps.^8^ ^,^ ^9^

*Bioinformatics analysis* - MS/MS spectra were selected from a database constructed from the *Lucilia* UniProt protein databank^17^ and the National Centre for Biotechnology Information (NCBI) GenBank database^18^ for determining the amino acid (aa) sequences; Proteome Discoverer 1.4 software (Thermo Fisher Scientific Inc) was used as search machine for protein identification. The ProtParam (Expasy) tool was used for calculating the physicochemical parameters,^19^is provided as a service to the life science community by a multidisciplinary team at the Swiss Institute of Bioinformatics (SIB ClustalW 2.0 for aligning the sequences^20^ and the PEP-FOLD3 server for predicting tertiary structure.^21^ The Antimicrobial Peptide Database (APD) (2016 version)^22^ was searched for predicting peptide parameters such as aa composition, residue percentage, hydrophobic ratio, protein-binding potential and sequence similarity.

The GalaxyHomomer server (1.0 version)^23^ was used for modelling oligomers; it calculates the interface area (in square angstroms) between a target chain and the other chains using the Naccess programme for calculating a molecule’s accessible area from a Protein Data Bank (PDB) format file and gives a docking score (a higher score is better). The docking score was calculated using an *ab initio* docking programme which predicts homo-oligomer structures considering the monomer structure based on the M-ZDOCK grid-based FFT docking method and ranks them as the top5 highest ranking clusters and the highest-score. The PEP-SiteFinder tool was used for scanning the sequences^24^ to determine peptide binding sites on protein surfaces and self-aggregation proficiency patterns.

*Peptide synthesis* - Four peptides were designed based on known sequences derived from the native AMPs used in this study. The Fundación Instituto de Inmunología de Colombia’s (FIDIC) Molecular Biology and Immunology Department chemically synthesised these four peptides’ sequences by solid phase peptide synthesis (SPPS) system, using Fmoc-protected amino acids (0.65 meq/g rink resin; IRIS Biotech). The peptides were hydrolysed with TFA/H20/TIS (95%/2.5%/2.5%) and purified by RP-HPLC; their purity and molecular weights were confirmed by MALDI-TOF and they were then lyophilised.^25^ Deionised water was used to dilute the peptides for evaluating antimicrobial activity and cytotoxic effects.

*Cell viability assays* - Cytotoxicity was evaluated against Vero cells (derived from monkey kidney epithelial cells). The cells were kept in Dulbecco’s Modified Eagle’s medium (DMEM), supplemented with 10% heat-inactivated bovine serum and antibiotic-antimycotic solution (100 units/mL penicillin, 100 g/mL streptomycin and 25 g amphotericin B) and incubated at 37ºC in a 5% CO_2_ atmosphere. A methyl thiazolyl tetrazolium (MTT) test was used for assessing cytotoxicity; 5x10^5^ cells/well were seeded in 96-well plates and incubated for 24 h. The cells were then incubated for 24 h with nine dilutions in a 1:2 ratio (starting from 250 μM) from each synthetic AMP and cell viability corresponding to peptide dilutions at 250, 31.25, 7.81 and 0.98 µM was plotted. Phosphate-buffered saline (PBS) and dimethyl sulfoxide (DMSO) added to wells containing growing cells and MTT were used as controls; these assays were performed in triplicate. The cells were subsequently incubated with 5 mg/mL MTT reagent for 4 h at 37ºC; the crystals which formed were dissolved with 150 µL isopropanol and absorbance was measured at 550 nm. The following formula was used for determining cytotoxicity: % viability = (cells treated with peptide/untreated cells) x 100.^9^

Antibacterial activity assay

*Bacterial strains* - The four bacterial strains used in this study (*S. aureus* ATCC 6538, methicillin-resistant *S. aureus* MRSA ATCC 43300, *E. coli* ATCC 26922 and the multi-drug resistant *P. aeruginosa* ATCC 1744 BAA) were obtained from the Universidad de La Salle and Universidad Antonio Nariño microbiology laboratories.

*Liquid and solid medium-based antibacterial assays* - These assays were used for estimating the fractions’ antimicrobial properties. *S. aureus* ATCC 6538 and *E. coli* ATCC 26922 were seeded on nutrient agar for 24 h before the inhibition assay in liquid medium. Seven bacterial colonies were suspended in 20 mL Mueller Hinton broth, thoroughly vortexed and optical density (OD) was measured at 620 nm. A Neubauer chamber was used for cell count, obtaining 1.5 x 10^8^ CFU/mL. This was followed by sowing on a 96-well plate (in triplicate) 100 μL Mueller Hinton medium (negative control), 20 μL 100 mg/mL gentamycin (Gram-negative bacteria) or penicillin-streptomycin (10,000U/mL-10,000μg/mL) (Gram-positive bacteria) with 80 μL bacteria (positive control), 100 μL medium with bacteria (growth control), 20 μL of each fraction with 80 μL bacteria and 20 μL unfractionated extract with 80 μL bacteria. The plate was incubated for 19 h at 37ºC, read at OD 620 nm wavelength and percentage growth calculated using the following formula: % survival = (450 nm Abs culture medium - 450 nm Abs filtrate or fraction extract / 450 nm Abs culture medium - 450 nm Abs bacterial suspension) x 100.^26^

*Minimum inhibitory concentration (MIC) measurements* - The assays for determining MIC were started from a concentrated 20 μL solution of each synthetic AMP, followed by dilutions at different concentrations (250, 125, 62.5, 31.25, 15.62, 7.81, 3.90, 1.95 and 0.97 μM). The four bacterial strains had been grown for 18-24 h at 37ºC in 5% CO_2_ and then 20 μL of each solutions was taken and mixed with 80 μL bacterial suspension (1.5 x 10^8^ CFU/mL) from each selected strain in a 96-well plate; 100 μL Mueller Hinton broth was used as growth control and 20 μL antibiotic mixed with 80 μL bacterial suspension as inhibition control. The plates were incubated for 18 h at 37ºC and absorbance was measured at 595 nm; the assays were carried out in triplicate. The MIC was defined as the lowest peptide concentration that completely inhibited growth.^27^

*Statistical analysis* - STATA software (version 12.0, Texas, USA) was used for all statistical analysis. One-way analysis of variance (ANOVA) was used for statistical comparison of combined treatment regarding cytotoxicity assays, with 95% confidence index and α = 0.05. Data has been presented as mean ± standard deviation (SD).

## RESULTS

*AMP purification and MS/MS characterisation* - *S. magellanica*-derived fat body filtrate extract had 4.15 mg/mL protein concentration. Regarding antibacterial activity, the filtered extract inhibited *S. aureus* ATCC 6538 and *E. coli* ATCC 26922 growth by 100%. The filtered extract had the same antibacterial effect as that of the evaluated antibiotics, penicillin-streptomycin for Gram-positive bacteria and gentamicin for Gram-negative bacteria.

**Fig. 1:**
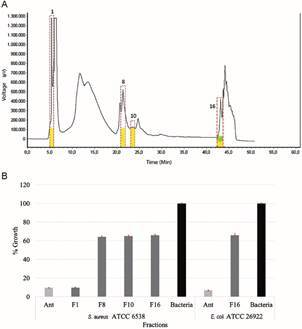
*Sarconesiopsis magellanica* larval fat body fractions’ chromatographic profile and antibacterial activity. (A) Chromatogram obtained by semi-preparative reversed-phase high-performance liquid chromatography (RP-HPLC) using a 0%-60% ACN/water/TFA solvent gradient. Twenty-three fractions were collected; fractions 1, 8 and 10 inhibited *Staphylococcus aureus* ATCC6538 growth (yellow) and fraction 16 inhibited *S. aureus* ATCC6538 (yellow) and *Escherichia coli* ATCC26922 growth (green). (B) Calculated percentage of growth of *S. aureus* and *E. coli* in liquid medium.

Fat body extracts were analysed by RP-HPLC; 23 eluted fractions having different retention times (RT) were manually collected. *S. aureus* ATCC 6538 had 9.57% growth (90.43% inhibition) regarding fraction 1 (RT 6.2 min) and fraction 8 (RT 21.1 min) and 64.90% for fraction 10 (35.1% inhibition: RT 23.6 min) (Fig. 1A-B). *S. aureus* and *E. coli* had 66.0% growth (34% inhibition: RT 43.9 min) regarding fraction 16 (Fig. 1A-B).

**Fig. 2:**
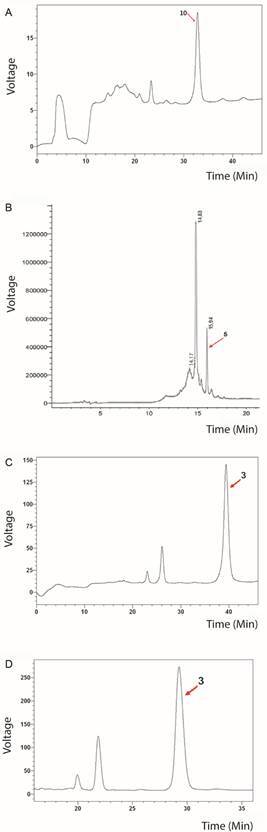
purifying fat body-derived chromatographic fractions. reversed-phase high-performance liquid chromatography (RP-HPLC) chromatography involved using an analytic column. (A) Fraction 1 having a 23%-28% ACN gradient in acidified water. The red arrow indicates fraction 10, denominated 1.10, eluted at 32.89 minutes and having antibacterial activity against *Staphylococcus aureus*. (B) Fraction 8; the ACN 0%-70% gradient was evaluated and fraction 5, denominated 8.5, is indicated by the red arrow had action against *S. aureus*. (C) Fraction 10 was purified using a 20%-30% ACN solvent gradient and antibacterial activity against Gram-positive bacteria was seen regarding fraction 3, denominated 10.3, indicated by the red arrow. (D) Fraction 16 was purified, having a 25%-35% ACN gradient. The red arrow indicates the fraction 3, denominated 16.3, which growth inhibited of *S. aureus* and *Escherichia coli.*

An analytical reversed-phase column was used for purifying the fractions having antibacterial activity against both bacteria; 11 fractions were collected from fraction 1, but only fraction 1.10 eluted at 32.89 min (Fig. 2A) inhibited *S. aureus* ATCC 6538 growth by 71.2%. Fractions 8 and 10 were further purified; fraction 8 resulted in seven fractions whereas fraction 10 resulted in three fractions. All fractions were collected and eluted at 15.94 min (fraction 8.5) (Fig. 2B) and 39.9 min (fraction 10.3) (Fig. 2C). RT inhibited Gram-positive bacteria growth by 40%. Three fractions were manually collected from purified fraction 16, at 17.51 min (fraction 16.1), 18.62 min (Fraction 16.2) and 21.3 min (Fraction 16.3) at RT (Fig. 2D). Fraction 16.3 was effective against *E. coli* ATCC 26922 and inhibited its growth by 61.9%.

**Fig. 3:**
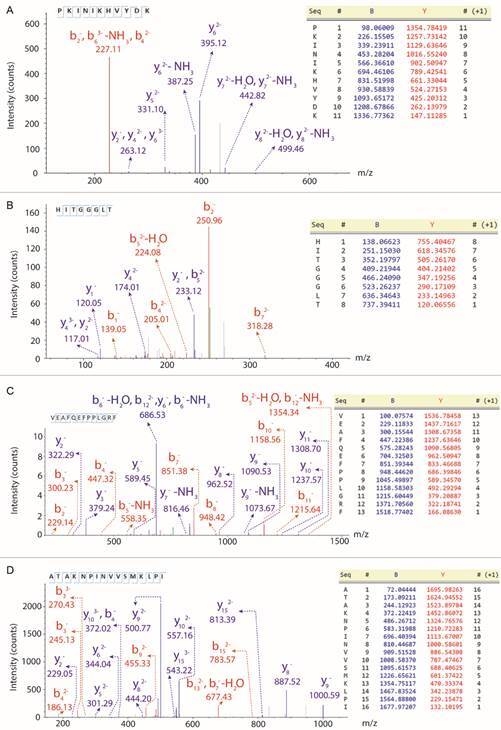
identifying peptides isolated from *Sarconesiopsis magellanica* fat body. Collision-induced dissociation (CID) spectra for de novo sequenced antimicrobial peptides (AMPs). Ions from -b (red) and -y (blue) series indicated in the upper part of the spectrum are from the peptides’ primary structure. (A) Sarconesin III (score 0.00). (B) Sarconesin IV (score 0.00). (C) Sarconesin V (score 3.01). (D) Sarconesin VI (score 0.00).

MS/MS analysis identified four molecules isolated from *S. magellanica* larval fat body called sarconesin III (Fraction 1.10), IV, (Fraction 8.5), V (Fraction 10.3) and VI (Fraction 16.3). Fig. 3A-D shows *de novo* MS sequence data for each peptide.

*Sequence analysis-based AMP characterisation* - Table I lists each AMP’s physicochemical properties. The hydrophilic/hydrophobic ratio for sarconesin III was 27 and it had a neutral net charge; its interaction with cell membranes was potentially weak. The eight-residue sarconesin IV which featured a triplet glycine motif (Gly-4, Gly-5 and Gly-6) was found to be the shortest peptide; it had a 25 hydrophilic/hydrophobic ratio. Sarconesin V had a 46 hydrophilic/hydrophobic ratio and contained negatively-charged residues, two prolines and one glycine residue. APD comparison predicted that this peptide lacked an alpha helix long enough for it to be considered an AMP. Sarconesin VI had a 50 hydrophilic/hydrophobic ratio, two lysine residues (positively-charged) and two prolines. These residues could interact with cell membrane according to the comparison in the APD.

**TABLE I:** Physiochemical properties of sarconesin peptides isolated from *Sarconesiopsis magellanica* fat body

Peptide	Sequence	Amount of aa	pI	Mass (Da)	Net charge	Hydrophilic/ hydrophobic ratio
Sarconesin III	PKINIKHVYDK	11	9.56	1,354.62	+2	27
Sarconesin IV	HITGGGLT	8	6.74	754.84	0	25
Sarconesin V	VEAFQEFPPLGRF	13	4.53	1,536.74	-1	46
Sarconesin VI	ATAKNPINVVSMKLPI	16	4.53	1,696	+2	50

aa: amount of amino acids; pI: isoelectric point; Da: Daltons (Predicted Mass).

**Fig. 4:**
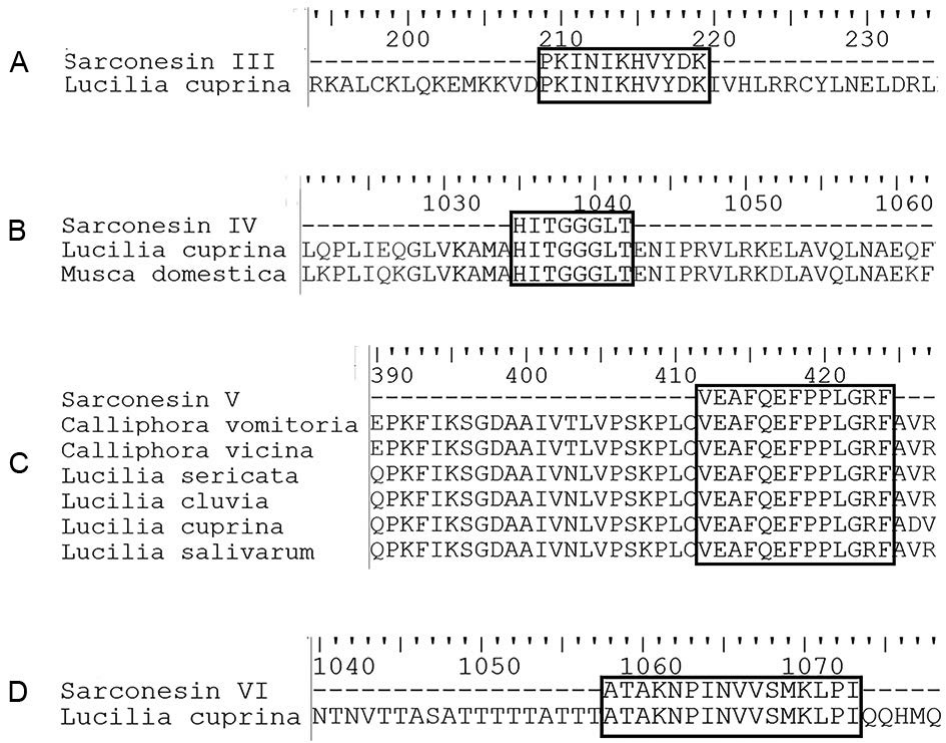
aligning sequences of peptides isolated from *Sarconesiopsis magellanica* fat body with sequences from other blow-fly species from the Calliphoridae family. (A) Sarconesin III. (B) Sarconesin IV. (C) Sarconesin V. (D) Sarconesin VI.

*Sarconesin sequence analysis* - A Proteome Discoverer database search revealed that sarconesin III occurs in a protein (NCBI XP_023300477.1) characterised from the blowfly *L. cuprina* (also from the Calliphoridae family (A0A0L0CMR4); BLAST results gave 100% identity (0.010 E-value) (Fig. 4A). Sarconesin III was positioned between residues 209 to 219 forming part of the GT1 subfamily (16-255 aa residues) located in the conserved MADF domain, similar to MYB, one of the trihelix GT transcription factors. The APD search showed that this peptide had 35.29% similarity with arthropods from the order Hymenoptera and 33.3% with annelids from the order Arhynchobdellida.

Sarconesin IV BLAST alignment gave 100% identity (7.3 E-value) with the *L. cuprina* (NCBI XP_0233305490.1) and *Musca domestica* (NCBI XP_005187874.1) trifunctional purine biosynthetic protein adenosine-3 (Fig. 4B). This protein has both molecular (e.g., ATP binding, phosphoribosyl-glycine ligase activity and metal ion binding, and biological functions (e.g., *de novo* IMP biosynthetics and purine nucleobase). This class of sarconesin forms part of this protein’s PurM C-terminal domain and PurM-like subfamily. The sarconesin IV sequence had 42.85% similarity with the species *Hylarana latouchi* (Order: Anura) and 40% with *Antherea mylita* (Order: Lepidoptera).

Sarconesin V had a similar sequence to a fragment from elongation factor 1-alpha 1 (eEF1a1) from blowfly specie from the Calliphoridae family reported in the UniProtKB database, such as *Calliphora vomitoria* (A0A221J5D0), *C. vicina* (E5BB32), *Lucilia sericata* (A0A064AKT1), *L. cluvia* (E5BB54), *L. cuprina* (E5BB60) and *L. salivarum* (E5BB75) (Fig. 4C). The BLAST search suggested that the peptide sequence had 100% identity (3e^-06^ E-value) with a bee species called *Melipona quadrifasciata* (access code NCBI KOX75059.1), also being similar to elongation factor 1, a translational elongation factor whose function is GTP binding. Sarconesin V formed part of the GTP_EFTU_D3 domain, accounting for 5.62% of the total *L. sericata* elongation factor sequence*.* It had 37.5% sequence similarity with the sequence of a peptide isolated from the myriapod *Scolopendra subspinipes subspinipes* reported in the APD database.

BLAST analysis revealed that sarconesin VI had a 16 aa-long conserved region, 100% identity (1e-08 E-value) with an *L. cuprina* non-characterised protein (XP_023294796.1) (Fig. 4D) and was similar to a protein containing the ANK_REP_REGION (A0A0L0CUM1) domain reported in UniProt. Its molecular function consisted of binding to protein phosphatase 2 A (PP2A); its biological functions were related to mitotic nuclear envelop reassembly and positive regulation of protein dephosphorylation.

Sarconesin VI was located at the C-terminal extreme between aa residues 1058 to 1073. The APD predicted that it had 40.9% homology with the codesane (COD) sequence, an AMP isolated from the bee *Colletes daviesanus* (AP02883) having activity against Gram-positive and Gram-negative bacterial growth and antifungal properties. This peptide had 35.29% to 40% similarity with other AMPs described for amphibians.

**Fig. 5:**
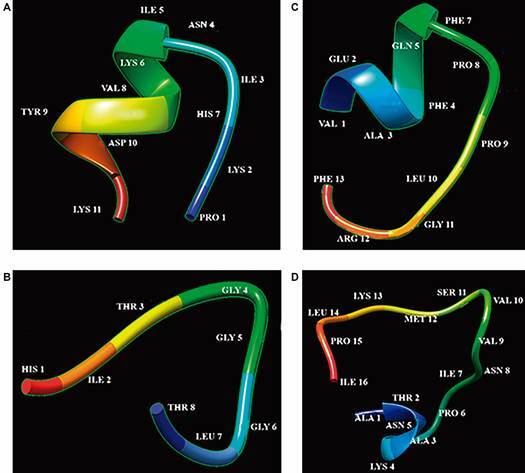
structure of sequenced *Sarconesiopsis magellanica* fat body-derived peptides. (A) Sarconesin III: α-helix type tertiary structure. (B - D) Sarconesin IV, V and VI: extended type tertiary structure.

**Fig. 6:**
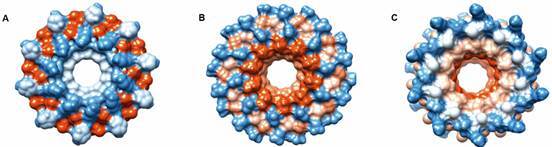
oligomer prediction of sarconesin. (A) Sarconesin III. (B) Sarconesin V. (C) Sarconesin VI. Figures represent a 10-mer subunits of each peptide.

*Predicting AMPs’ aggregation mechanism* - PEP-FOLD3 was used for predicting AMP tertiary structure. Sarconesin III had an α-helix structure (Fig. 5A), whilst sarconesin IV (Fig. 5B), V (Fig. 5C) and VI had linear type structures; 37.5% of the sarconesin IV aa sequence consisted of glycine residues (Fig. 5D). Peptide-peptide interactions through a 10-mer oligomer were predicted, suggesting a possible MoA. Sarconesin III had a 4,228.7 Angstrom^2^ interface area and a 398.596 docking score (Fig. 6A). Sarconesin V had a well-defined oligomer, 550.654 docking score (highest) and 3,248.4 Angstrom^2^ (Fig. 6B); sarconesin VI had a significant cavity (5,749.9 Angstrom^2^) and 382.38 docking score (Fig. 6C). Oligomer interactions could not be predicted for sarconesin IV due to its short sequence.

**Fig. 7:**
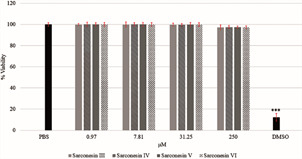
cell viability effects of sarconesins incubated at different concentrations with Vero cell line. ***: highly significant statistical difference with a p < 0.001.

*Vero cell viability* - Synthetic AMP (sarconesin III, IV, V and VI) effects on Vero cell viability was tested for understanding these molecules’ cytotoxicity. The AMP concentrations evaluated here revealed 97% to 99% cell viability; the opposite effect was observed in DMSO-treated cells (12% viability), having highly significant differences (p < 0.0001) when comparing the concentrations evaluated here (Fig. 7).

*Minimum inhibitory concentration* - Synthetic AMP (i.e., sarconesin III, IV, V and VI) inhibitory activity against Gram-positive and Gram-negative bacteria was evaluated; Table II lists the MICs for each AMPs. It was observed that sarconesin VI was effective against the four bacteria evaluated here, whilst the other two AMPs exclusively inhibited *S. aureus* and *S. aureus* MRSA growth. It was found that a higher peptide concentration was required to inhibit resistant bacteria (i.e., *S. aureus* MRSA and *P. aeruginosa* BAA) by 100%, regardless of the peptide.

**TABLE II:** Minimum concentration for inhibiting Gram-positive and Gram-negative bacterial growth by 100%

Bacteria	MIC peptide (μM)			
	Sarconesin III	Sarconesin IV	Sarconesin V	Sarconesin VI
^*^ *Staphylococcus aureus* ATCC 6538	4.3	5.1	19.6	2.1
^*^ *S. aureus* MRSA ATCC 43300	171.8	182.9	-	141.3
^**^ *Escherichia coli* ATCC 26922	-	-	-	2.7
^**^ *Pseudomonas aeruginosa* BAA ATCC 1744	-	-	-	113.9

MIC: minimum inhibitory concentration; *: Gram-positive; **: Gram**-**negative; -: MIC not detected.

## DISCUSSION

Insects’ fat body is the main organ for AMP synthesis and production in response to microbial attack. The fat body extracts obtained from filtration inhibited *S. aureus* and *E. coli* growth; the same effect has been reported regarding *S. magellanica* and *L. sericata* larval fat body extracts for treating diabetic rabbits’ wounds infected by the same bacteria^15^ and *C. vicina* larval fat body which has also had an effect against Gram-positive and Gram-negative bacteria.^14^ Such results could confirm that pathogens (i.e., bacteria) activating the innate immune system lead fat bodies to synthesise AMPs, thus becoming a therapeutic alternative for countering the antimicrobial resistance which has increased alarmingly worldwide, causing costs predicted to be in excess of 100 billion USD by 2050.^28^

Four peptide sequences were identified and characterised from *S. magellanica* larval fat body; however, these were not similar to AMPs isolated from the same blowfly’s ES from which sarconesin^8^ and sarconesin II had been obtained.^9^ This could be explained by the fact that once the larval fat bodies containing all the genes encoding AMPs in response to microorganism invasion have been produced,^29^ they become released to different tissues and each could produce their own *de novo* AMPs. This could account for the lack of homology which has already been studied in *Drosophila melanogaster*.^30^

Physicochemical properties such as net charge, hydrophobicity, amphipathicity, sequence, size and structure are fundamental factors for determining antibacterial activity and toxicity.^31^ Sarconesin III was found to have a 1,354 Da, α-helix structure, a +2-net charge and to be soluble in water. This peptide’s cationicity could enable it to electrostatically bind to Gram-positive and Gram-negative bacterial membranes consisting of predominant negatively-charged molecules, such as phospholipid heads.^32^ AMPs could be inserted into bacterial membrane (due to their α-helix structure), leading to its thinning and thereby causing its disruption.^33^ Sarconesin IV had high glycine residue content in its structure; it has been reported that glycine-rich AMPs are cationic.^34^ This *S. magellanica* larval fat body-isolated AMP had 0 net charge, resembling a hydrophobic molecule, and its predicted tertiary structure suggested an extended type. This peptide had a glycine-rich region which might have been related to its higher molecular flexibility due to sidechain size and degrees of backbone freedom, leading to structural versatility regarding peptide conformation.^35^ Little has been reported regarding peptide dynamics (i.e., chemistry, structure-activity relationship and properties) for insect peptides having such composition.^36^

Regarding consensus mechanism, sarconesin might form self-aggregations upon microbial membrane contact (as predicted for fraction II), similar to the *Antheraea mylitta* AMP. This peptide has been shown to contain a Gly-rich peptidylglycine α-amidating monooxygenase (PAM) promoting lipid-interactions with the 1-palmitoyl-2-oleoylphosphoethanolamine (POPE) bilayer’s lipid aliphatic chain and be linked to membrane distortion.^36^

Sarconesin V was shown to have 1,536.74 Da and -1 net charge. Anionic peptides have been little studied in insects; three peptides have been reported to date. Two of them having anti-Gram-positive activity were isolated from *Galleria mellonella* larval hemolymph (known as Gm1 and Gm2).^37^ MDpep5 isolated from *M. domestica* larvae had activity against both Gram-positive and Gram-negative bacteria.^38^

The coupling punctuation suggested strong peptide-peptide interaction targeting the cell wall; such synergism between peptides has been previously proposed regarding temporin L which has a pore-forming mechanism inducing a membrane thinning effect.^39^ Sarconesin VI was found to be a cationic peptide; its structure consisted of an α-helix and it has been classified as an extended peptide.^40^ Our peptide oligomer model suggested that surface-exposed residues have a strong tendency to chain aggregations stabilised by peptide binding sites and hydrophobic interactions contacting cell membranes and peptide oligomeric state based on the GalaxyHomomer tool wich included an accurate prediction based on template-based modeling and ab-initio docking improving quality models.^24^ The best oligomer model is predicted upon a sequence and structure-based oligomer template detection, ab initio docking, oligomer building, loop/terminus modeling, and overall refinement.

The aforementioned physicochemical properties are important for defining a peptide’s activity and such activity depends on the set of these functions; nevertheless, there is still no definite rule regarding the perfect amount of hydrophobic or charged residues for boosting activity and reducing cytotoxicity as this varies from peptide to peptide.^41^ Some authors have found that a +8 or +9 increase in net charge has significantly increased haemolytic activity and that this occurs due to transmembrane channels and pores forming in eukaryotic cells;^42^ increased hydrophobicity has been related to a toxic effect produced by AMPs binding to membranes.^41^

Peptide toxicity regarding mammalian cells represents a limitation for producing and using peptides in clinical applications.^43^ Sarconesin III, IV, V and VI cytotoxicity was evaluated in the present study; it was found that different synthetic AMP dilutions had no toxic effect on Vero cells. Low cytotoxicity evaluated *in vitro* in mammalian cells has been documented for different dipteran peptides, e.g., L-serCecs 1-6, isolated from *L. sericata,* sarcotoxin IA, B and C (*Sarcophaga peregrina*), stomoxyn (*Stomoxys calcitrans*), Mdc (*M. domestica*) and Cec peptide A and Cec B (*D. melanogaster*, *Culex pipiens* and *Aedes albopictus*).^44^ Almost 100% viability has been observed in *Hirudo medicinalis*-treated cells, not differing significantly from that of unexposed control cells^45^ and designed peptides. RR and RR1 had low cytotoxicity regarding HaCat and J774.1A and no cytotoxicity was reported at 256 μM, this being the highest concentration evaluated.^43^

Synthetic sarconesin isolated and characterised from *S. magellanica* larval fat body inhibited Gram-positive and Gram-negative bacteria growth; however, sarconesin III, IV and V specifically inhibited *S. aureus* and *S. aureus* MRSA Gram-positive growth whilst sarconesin VI acted against the four bacteria evaluated here. This differentiation regarding sarconesin ability to inhibit bacterial growth has been reported in necrophagous blowfly peptides having broad spectrum activity, i.e., lucifensin,^5^ FLIP7,^7^ sarconesin^8^ and sarconesin II,^9^ or peptides only acting against Gram-positive bacteria, such as phormicin A,^46^ or Gram-negative bacteria such as diptericin A (DptA).^47^ Cecropins having exclusive antibacterial activity have been described for Gram-positive or Gram-negative bacteria in *M. domestica*, as have cecropins having broad spectrum activity.^48^

*S. aureus* and *E. coli* MICs were low, ranging from 1.2 to 17.2 μM; such range agreed with MIC ranges reported for antimicrobial peptides isolated and characterised from different species, for example, *M. domestica* cecropin MICs ranged from 0.5 to > 64 μM against *E. coli* and *S. aureus*.^48^ *Hirudo medicinalis*-isolated peptides that inhibited *Bacillus subtilis* and *E. coli* bacterial growth had MICs ranging from 1.5 to 102 μM.^49^ *Lasioglossum laticeps venom*-isolated LI, L-II and L-III peptides acted against *S. aureus* (3.9 to 14.3 μM MIC), *E. coli* (1.4 to 1.7 μM MIC) and *P. aeruginosa* (14.4 to 18.7 µM MIC).^50^ However, sarconesin MICs required to inhibit *S. aureus* MRSA and *P. aeruginosa* BAA resistant bacteria growth by 100% were notably higher (141.3 to 182.9 μM MIC), similar to the MIC reported for RR (128 μM) and RR1 (256 μM) designed as small sequence peptides.^43^ Needing a higher peptide (sarconesin) concentration for inhibiting bacterial growth could have been related to their intrinsic refractory characteristics regarding their resistance.

A feasible strategy for enhancing sarconesin effect in future work would be to combine synthetic AMPs with conventional antibiotics which have given excellent results; for example, Almaaytah et al.,^51^ demonstrated this combination’s powerful synergistic activity against resistant bacteria in recent work involving standard experimental tests. Specific time-kill assays established that none of the antimicrobial agents used individually (i.e., conventional antibiotics and AMPs) could induce microorganism cell death within 24 h of exposure; however, only a 4-hour interval was needed to significantly reduce resistant strain. *S. aureus* (ATCC 29213) count and an 8-hour one for *S. aureus* ATCC (33591). The complete eradication of resistant Gram-negative strain *P. aeruginosa* ATCC (BAA2114) was achieved after 8 h exposure to the indicated combinations’ synergistic action.

*In conclusion* - This study involved isolating and characterising *S. magellanica* larval fat body derived-AMPs. Only four of a set of fat body-derived fractions (1.10, 8.5, 10.3 and 16.3) were identified by MS/MS analysis, following a selective process of purification and antibacterial evaluation (i.e., sarconesin III, IV, V and VI). These AMPs were characterised according to their physicochemical properties; proteomic databases were searched using sequence analysis to align these molecules in proteins’ specific sites, mostly belonging to Diptera species and many of them linked to the Calliphoridae family. Bioinformatics tools were used for determining percentage similarity and identity with these proteins and the AMPs’ tertiary structure was predicted (except for sarconesin VI).

Sarconesin molecules were synthesised from native AMPs. MIC-based antibacterial evaluation highlighted these AMPs’ efficient action against Gram-positive bacteria and, to a lesser extent, against Gram-negative and drug-resistant bacteria. None of the synthetic AMPs had a cytotoxic effect on Vero cells.

It has been demonstrated that *S. magellanica* larval fat bodies are an important source of AMPs and such molecules can be extremely useful regarding antimicrobial therapy for treating wounds infected by various microorganisms and combating various types of infectious diseases and antimicrobial resistance.
